# COMSOL Multiphysics® modelling of oxygen diffusion through a cellulose nanofibril conduit employed for peripheral nerve repair

**DOI:** 10.1186/s12938-021-00897-1

**Published:** 2021-06-15

**Authors:** Julia Towne, Nicklaus Carter, David J. Neivandt

**Affiliations:** 1 Department of Chemical and Biomedical Engineering, University of Maine, Orono, ME 04469 USA; 2 Graduate School of Biomedical Science and Engineering, University of Maine, Orono, ME 04469 USA; 3 Forest Bioproduct Research Institute, University of Maine, Orono, ME 04469 USA

**Keywords:** Cellulose nanofibril (CNF), Peripheral nerve injury and repair, Oxygen diffusion, Neural conduit, COMSOL Multiphysics® modelling

## Abstract

**Background:**

Peripheral nerve injury can cause significant impairment, and the current methods for facilitating repair, particularly over distances greater than approximately 1 mm, are not entirely effective. Allografts, autografts, and synthetic conduits are three of the most common surgical interventions for peripheral nerve repair; however, each has limitations including poor biocompatibility, adverse immune responses, and the need for successive surgeries. A potential new method for promoting peripheral nerve repair that addresses the shortcomings of current interventions is a biocompatible cellulose nanofibril (CNF) conduit that degrades in-vivo over time. Preliminary testing in multiple animal models has yielded positive results, but more information is needed regarding how the CNF conduit facilitates nutrient and gas flow.

**Results:**

The current work employs 3D modelling and analysis via COMSOL Multiphysics® to determine how the CNF conduit facilitates oxygen movement both radially through the conduit walls and axially along the length of the conduit. Various CNF wall permeabilities, conduit lengths, and nerve-to-conduit diameter ratios have been examined; all of which were shown to have an impact on the resultant oxygen profile within the conduit. When the walls of the CNF conduit were modeled to have significant oxygen permeability, oxygen diffusion across the conduit was shown to dominate relative to axial diffusion of oxygen along the length of the conduit, which was otherwise the controlling diffusion mechanism.

**Conclusions:**

The results of this study suggest that there is a complex relationship between axial and radial diffusion as the properties of the conduit such as length, diameter, and permeability are altered and when investigating various locations within the model. At low wall permeabilities the axial diffusion is dominant for all configurations, while for higher wall permeabilities the radial diffusion became dominant for smaller diameters. The length of the conduit did not alter the mechanism of diffusion, but rather had an inverse relationship with the magnitude of the overall concentration profile. As such the modeling results may be employed to predict and control the amount and distribution of oxygenation throughout the conduit, and hence to guide experimental conduit design.

## Background

**Fig. 1:**
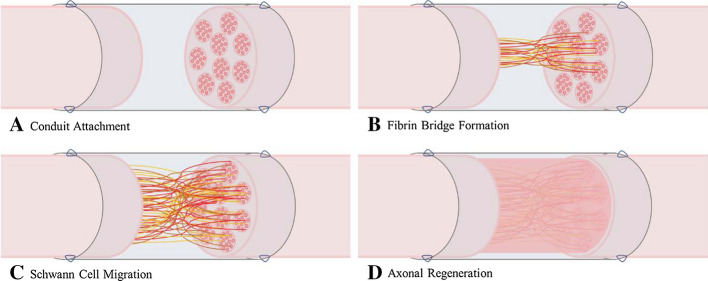
Four basic steps of peripheral nerve regeneration within a synthetic conduit

Peripheral nerves have the innate ability to regenerate over short distances. The process entails Wallerian degeneration, which begins shortly after nerve transection and initiates eventual regeneration [1]. Briefly, an influx of calcium to the injury site initiates degeneration of the distal stump by activating the protease calpain [1, 2]. In addition to laying down myelin during the regeneration phase, during the degeneration process Schwann cells phagocytose debris and produce growth factors [3], which leads to macrophage recruitment for further phagocytosis and commencement of nerve regeneration [1]. Regeneration occurs from a growth cone on the proximal stump along bands of Büngner, formed by Schwann cells that bridge the gap to the distal stump [4]. While innate peripheral nerve repair does occur it has limited capabilities, and interventional methods such as neural grafts and conduits may be necessary when damage is too severe [5]. While these methods can be effective, there are many issues that may arise during surgery and after implantation [6]. Common issues include biocompatibility, rigidity, successive surgeries for removal of synthetic conduits, as well as immune responses caused by foreign bodies [7]. Autologous nerve grafts minimize the potential of an immune response as they come from the same individual, but they result in the loss of nerve tissue in a secondary location and are in limited supply [8]. Allografts conversely are in greater supply, but require a significant immunosuppressant regimen to avoid rejection of the foreign tissue [8]. Most current conduits are constructed of synthetic materials, and since some compositions do not degrade over time (i.e., silicone) they must be removed due to patient discomfort, thereby requiring a second potentially unnecessary procedure [9]. Figure 1 presents the generalized step-by-step process of nerve regeneration within a conduit, after which the conduit may need to be surgically removed [10].

A potential solution to these problems, and one explored by the authors and their collaborators, is the use of cellulose nanofibril (CNF) based conduits. Such conduits have been shown to be biocompatible, flexible, and promote peripheral nerve growth. The general mechanism of regeneration is believed to follow that presented in Fig. 1, after which the conduit is intended to degrade rather than require removal. In preliminary unpublished work, two rounds of testing of the CNF conduits in a murine sciatic nerve model have shown significantly improved nerve regeneration in comparison to control groups without a conduit. While the in vivo test results are promising, little is known regarding which details of the conduit construction are beneficial for regeneration and may potentially be further optimized, and conversely which details of the conduit may potentially be detrimental for regeneration and should be minimized or omitted. Of particular interest is the effect of the conduit on the diffusion of molecules known to be beneficial, and conversely detrimental, to peripheral nerve regeneration, for example oxygen, calcium, glucose, and carbon dioxide [11, 12]. Conduit parameters considered likely to be relevant to molecular diffusion include length, wall permeability and nerve to conduit diameter ratio. Indeed, the murine studies referenced above demonstrated a significant difference in recovered grip strength between groups implanted with different length conduits over a consistent nerve gap. While there are currently no data showing differences in functional recovery of animals when the ratio between the nerve and the conduit diameter are changed, it may be expected that such a change would influence the distribution and extent of diffusion and, therefore, the effectiveness of the conduit. Obtaining such predictive data is necessary to determine the optimal conditions for nerve regrowth and further understand, as well as advance, conduit design.

A critical aspect of conduit performance is to ensure that diffusion of pro-regenerative molecules into the interior of the conduit is facilitated, while diffusion of waste materials out of the conduit is promoted [11]. For the current work the primary molecule of interest was oxygen, arguably the most critical chemical species required for homeostasis. In addition, it is noted that while necessary for successful nerve regeneration, the detailed role of oxygen in nerve regeneration is unclear [13, 14]. For example, Cho et al. found that intermittent hypoxia may be beneficial to nerve regrowth as the lack of oxygen triggers the activation of Hypoxia-Inducible Factor (HIF) [15]. When activated, this transcriptional mediator recruits co-activators and modifies the chromatin structure of the injured nerve, which controls gene expression for the transcriptional response. It was discovered that the absence of HIF resulted in impaired nerve regeneration, suggesting that hypoxia may potentially enhance nerve regeneration [15]. In addition, Yao et al. have shown that hypoxic conditions indirectly improves neural regeneration by enhancing cell migration to the injury, particularly Schwann cells [16].

Conversely, other studies indicate that hyperbaric oxygen (HBO) therapy (inducing hyperoxygenation) may be an effective treatment for nerve injuries [17, 18]. Indeed HBO studies employing a murine sciatic nerve model demonstrated greater nerve regeneration with less evidence of edema, coupled with enhanced conservation of cytostructural features vs. controls [19]. HBO is a method of treatment that has been employed for more than 30 years [19], and promotes regeneration through hyperoxygenation as well as several secondary mechanisms. For example, increased oxygen concentration is known to be correlated with increased ATP and GTP levels. HBO is also known to reduce the inflammatory response and aids in the conservation of healthy tissue by reducing oxidative stress and preventing apoptosis [19]. Interestingly, Lim et al. have demonstrated that the regenerating proximal nerve consumes approximately twice as much oxygen as a healthy nerve, supporting the concept that elevated concentrations of oxygen may be beneficial [20]. Clearly such findings are, however, contrary to those indicating that hypoxic conditions support repair, and highlight the need for a greater understanding of the role of oxygen in nerve regeneration.

## Results and discussion

**Fig. 2:**
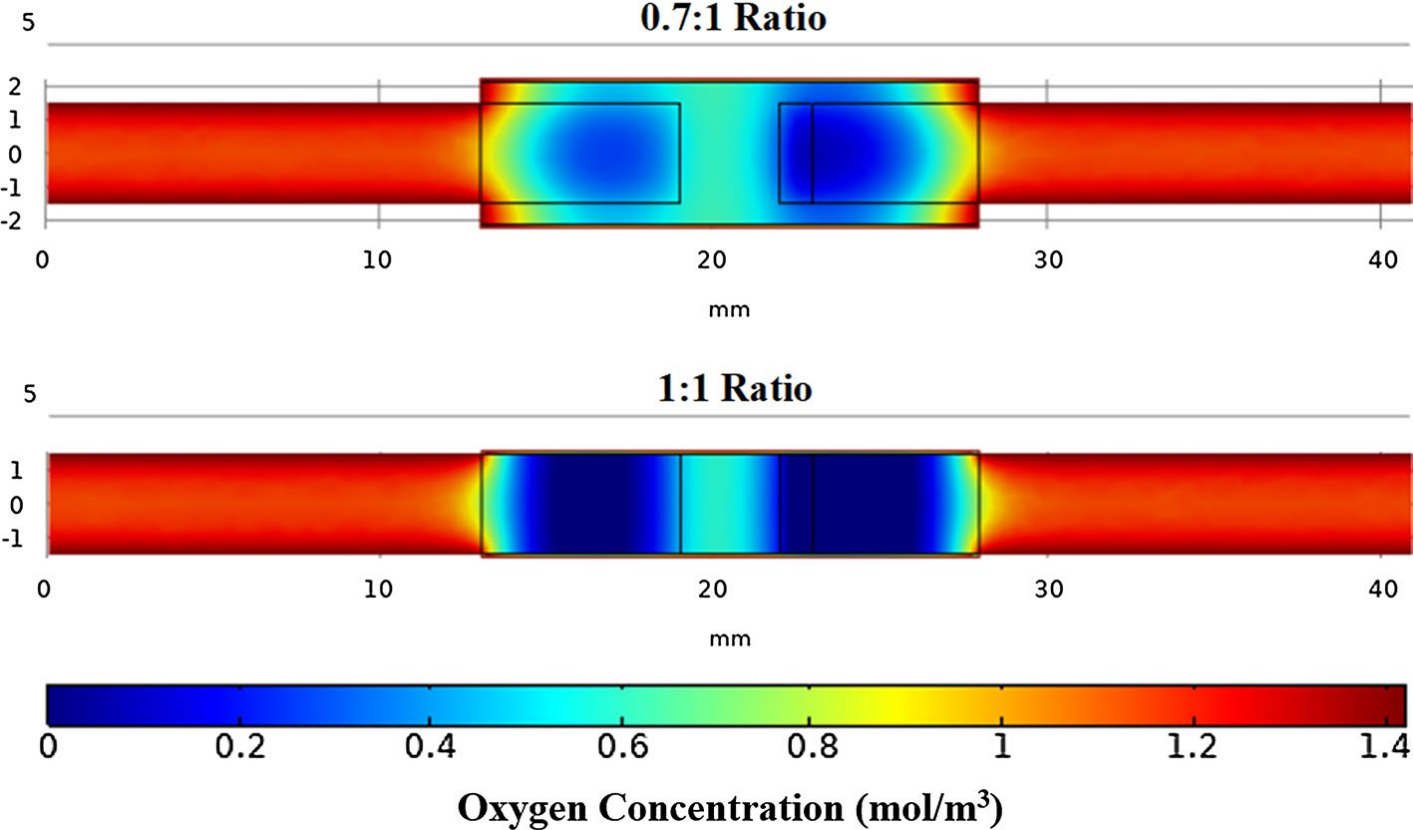
Oxygen concentration profiles for a 0.7:1 and 1:1 nerve to conduit diameter ratio. The conduit was 15 mm long and the model was run for 30 min and 50 min, respectively

One of the major variables of interest was the effect of nerve to conduit diameter ratio on oxygen concentration profiles. To evaluate the effects, the model was run at the six different nerve to conduit ratio values over the time period required to reach equilibrium, or until oxygen concentrations at location 1 reached the minimum possible value of 0. A range of nerve to conduit diameter ratios of 0.7:1 to 1:1 was selected to mimic that employed in the previously mentioned unpublished in vivo murine studies. Figure 2 presents the final oxygen concentration profile of the model run for the lowest and highest nerve to conduit diameter ratios and is presented as an axial-cut plane on a continuous color scale of blue (0 mol/m^3^) to maroon (1.4 mol/m^3^).

**Fig. 3:**
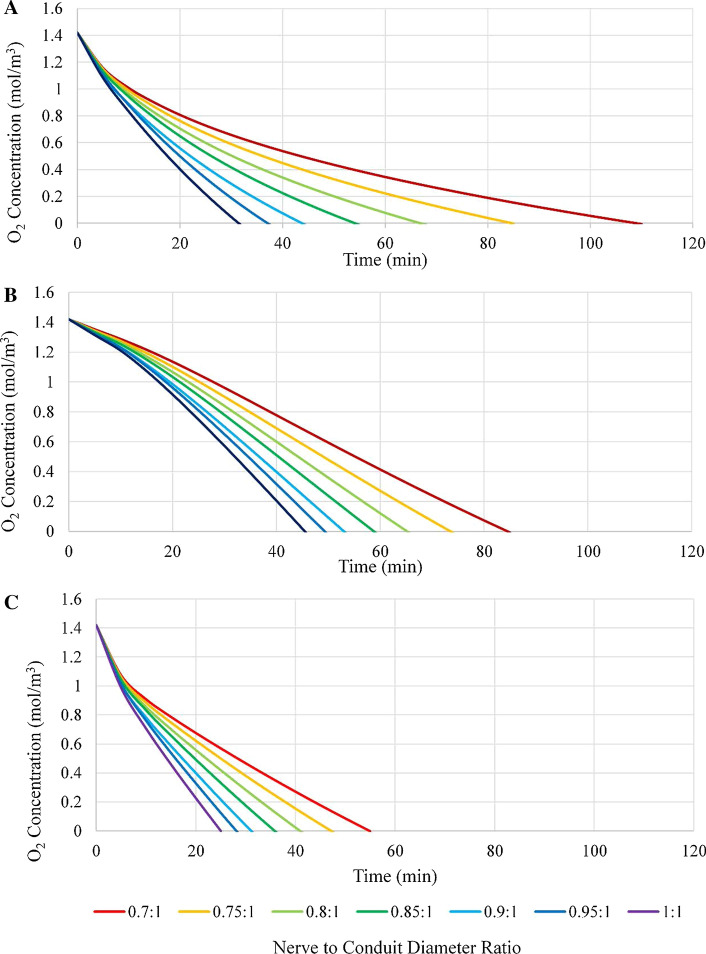
Oxygen concentration vs. time graphs for nerve to conduit diameter ratios of 0.7:1, 0.75:1, 0.8:1, 0.85:1, 0.9:1, 0.95:1, and 1:1 at **a** location 1, **b** location 2, and **c** location 3

It may be seen from investigation of Fig. 2 that regions of lowest oxygen concentration were located toward the end of the nerve stumps, and that the lowest overall concentration was at the interface of the regenerating region and the baseline region of the proximal stump, where oxygen consumption is twice that of baseline nerve consumption. It is further evident from Fig. 2 that varying the ratio of the nerve to conduit diameter does not change the general oxygen distribution profile; however, the actual oxygen concentration at given points is affected. Specifically, lower oxygen concentrations are observed within the conduit for higher nerve to conduit diameter ratios (that is, when the conduit diameter approaches the nerve diameter), a fact likely attributable to decreased axial diffusion due to the reduced volume of ISF. The oxygen concentration in the center of the nerve gap is greater than toward the ends of the nerve stumps, an observation which is consistent with the lack of consumption. At the ends of the conduit, where the nerve stumps transition from being enclosed to non-enclosed, the oxygen concentration is observed to rapidly increase to that of the exterior oxygen concentration present in the ISF. The qualitative representation of the oxygen concentration data of Fig. 2 may be presented in a quantitative manner employing the three locations described above and plotting oxygen concentration vs. time at each, see Fig. 3.

**Fig. 4:**
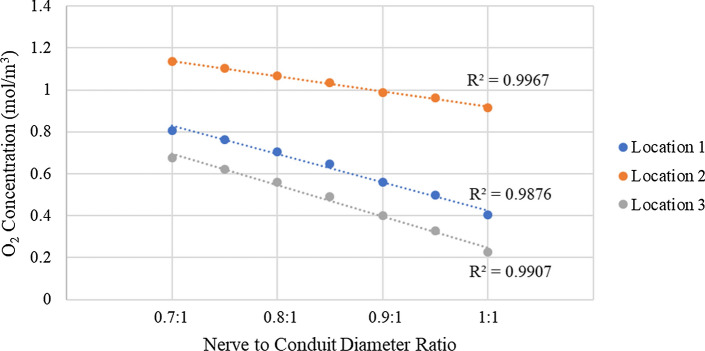
Oxygen concentration vs. nerve to conduit diameter ratio at t = 25 min for all 3 locations

Investigation of the graphs of Fig. 3 reveals several interesting attributes of the model and its relationship with conduit oxygen permeability. First, it is noted that at location 3 the oxygen concentration for all nerve to conduit diameter ratios reached a concentration of 0 mol/m^3^ in the shortest time period of the three locations (under 60 min for all nerve to conduit diameter ratios), a finding that is consistent with the observation from the qualitative data of Fig. 2 that the oxygen concentration at location 3 was lower than at locations 1 and 2. In addition, analysis of the concentration vs. time curves of 6 (a), (b) and (c) indicates that the curves for locations 1 and 3 are of similar shape, although of different scale, with a rapid, near linear, initial decline followed by a transition to a more gradual decay. Conversely the curves for location 2 have a shallower initial decline followed by a transition to a slightly more rapid decay. For each location a clear trend in the oxygen concentration at a given time as a function of the nerve to conduit diameter ratio is evident, with concentration decreasing at all locations as nerve to conduit diameter increases. Figure 4 presents a plot of oxygen concentration vs. nerve to conduit diameter ratio at an arbitrary timepoint of 25 min.

**Fig. 5:**
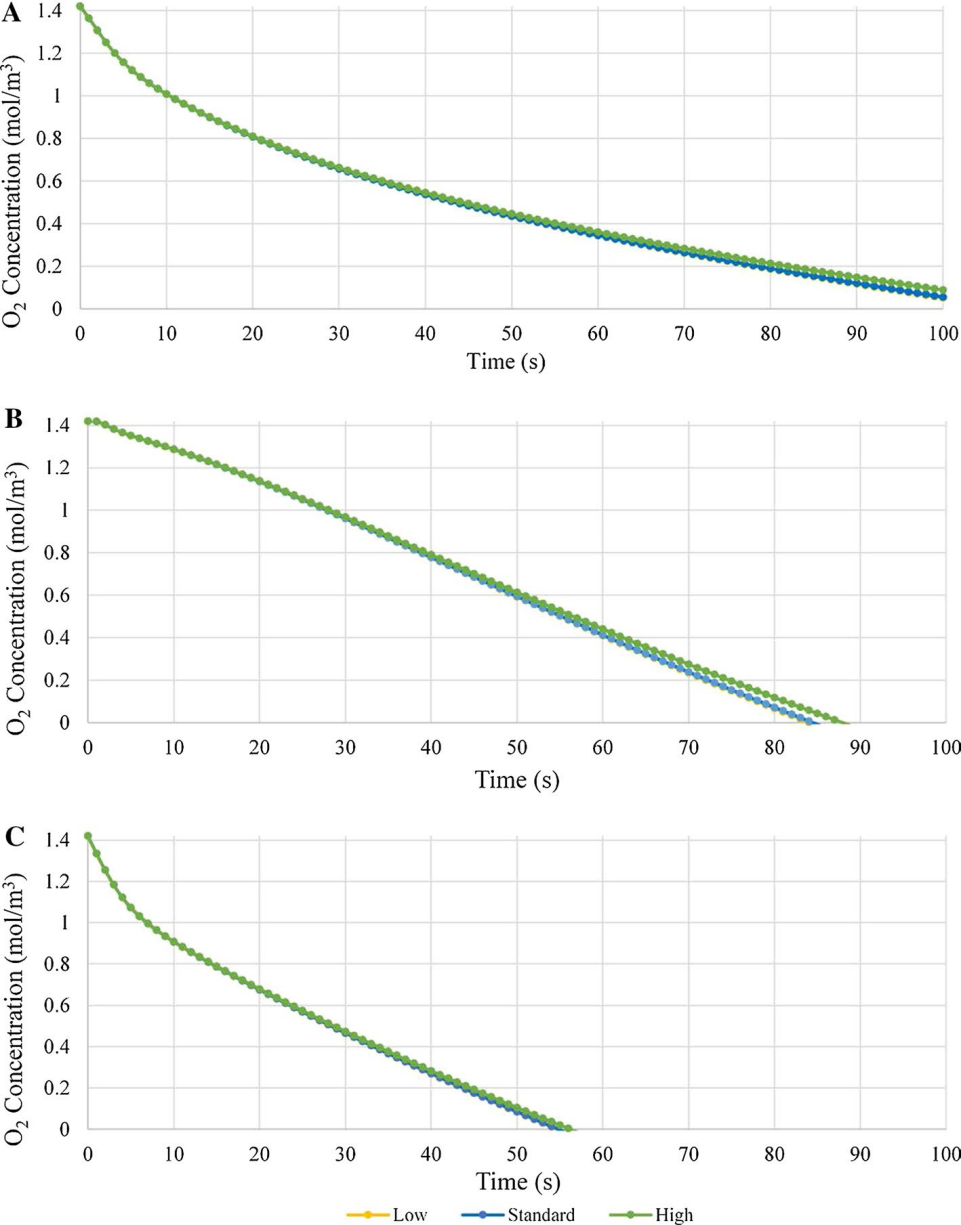
Oxygen concentration vs. time plots from t = 0 min to t = 100 min employing a CNF oxygen permeability value as per Table 2 (standard), a permeability one order of magnitude lower (low), and a permeability one order of magnitude higher (high). Shown for **a** location 1, **b** location 2, and **c** location 3

From investigation of Fig. 4, it is evident there is a relatively linear relationship between decreasing oxygen concentration and increasing nerve to conduit diameter ratio at all locations. Location 2 has the highest overall oxygen concentration, a fact attributable to the lack of oxygen consumption in the region between the two nerve stumps. Location 3, conversely, has the lowest overall oxygen concentration, an observation that is consistent with the proximal stump terminating with a region that consumes twice the oxygen of a non-regenerating nerve. The observed relationship of decreasing oxygen concentration within the conduit with increasing nerve to conduit diameter ratio may be understood via consideration of the resultant decrease in luminal ISF volume that results from a more closely matched nerve and conduit diameter (i.e., greater nerve to conduit diameter ratio). Specifically, as the nerve to conduit diameter ratio increases, the distance between the nerve and the conduit inner wall (filled with ISF) decreases and hence the cross-sectional area through which oxygen within the ISF can diffuse axially into the conduit decreases, leading to lower oxygen concentrations at locations within the conduit. The high linearity of the relationship between oxygen concentration and nerve to conduit diameter ratio suggests that there is very little influence from radial diffusion of oxygen through the walls of the conduit (or that it is invariant with nerve to conduit diameter ratio). To test the sensitivity of the model predictions to the permeability of the conduit walls to oxygen, simulations were run employing CNF permeabilities one order of magnitude above and one order of magnitude below the value listed in Table 2 in the Methods section. The results of this analysis are presented in Fig. 5 as plots of oxygen concentration vs. time at the three locations within the conduit employing CNF permeabilities spanning two orders of magnitude.

Investigation of Fig. 5 reveals that varying the oxygen permeability of the CNF conduit walls by two orders of magnitude has only a minimal effect on the oxygen concentration within the conduit. As such, it is concluded that the value employed for the oxygen permeability of CNF in the present work is likely sufficiently accurate to give the COMSOL Multiphysics® model reliable predictive power. It is noted, however, that the majority of the model predictions trend to zero oxygen concentration within the conduit in a timeframe that is very short (typically hours) relative to the implant durations (weeks–months), a continuously hypoxic scenario that is likely not favorable for nerve regeneration in-vivo. It was, therefore, determined that simulations should be run with significantly greater conduit wall permeability to verify the veracity of the model itself, and to provide insight into how radial diffusion pathways affect the resultant oxygen concentration profile when employing conduits with greater wall oxygen permeability.

**Fig. 6:**
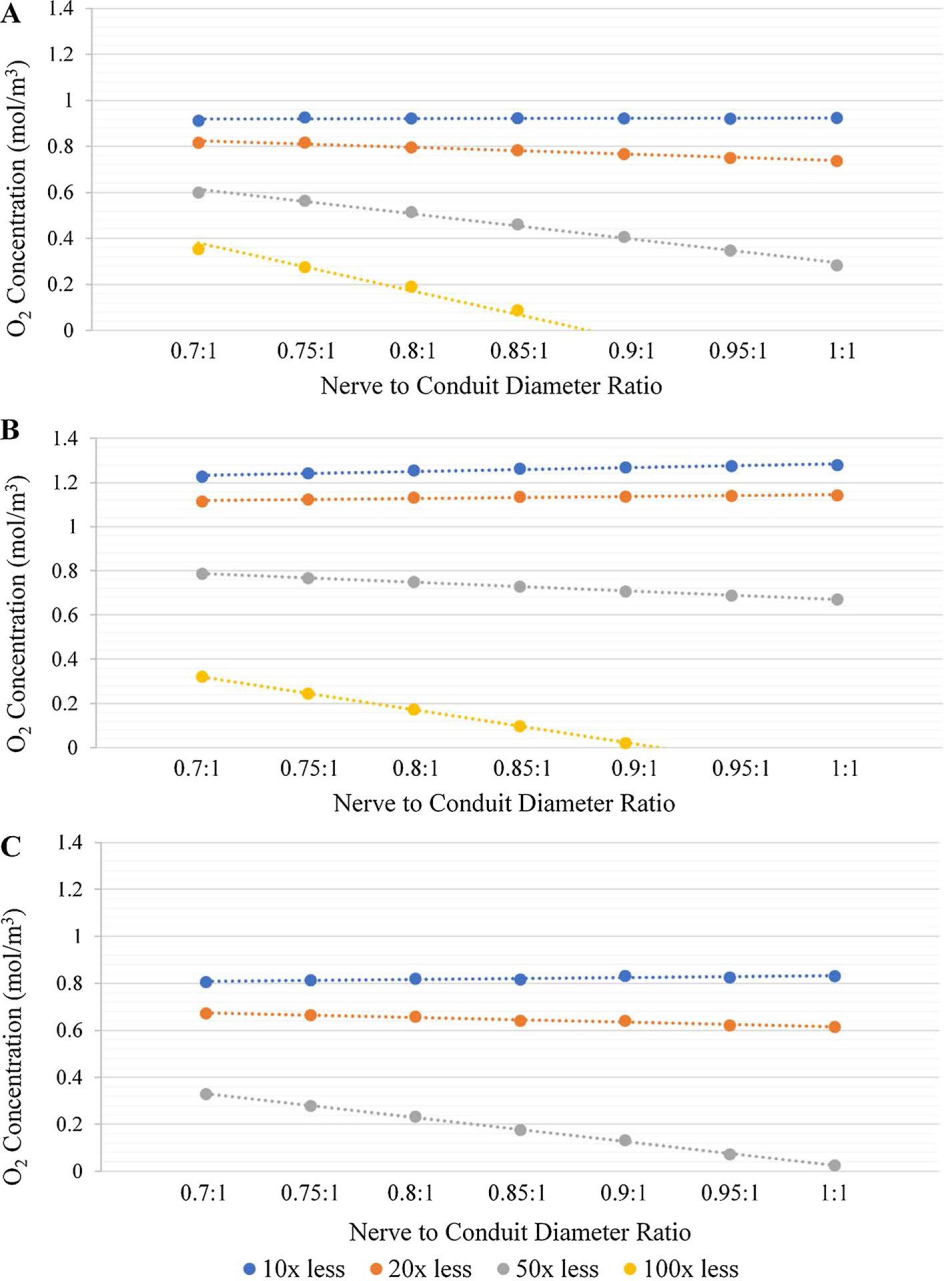
Oxygen concentration vs. nerve to conduit diameter ratio at locations 1 (**A**), 2 (**B**) and 3 (**C**) for varying conduit wall oxygen permeabilities of 10 (blue), 20 (orange), 50 (grey) and 100 (yellow) times less than ISF

To determine an appropriate range for testing the effect of the oxygen permeability of the conduit wall on oxygen concentrations within the conduit, the permeability of materials currently used for conduit construction were reviewed, and related to the limiting case of a conduit wall with the permeability of the surrounding media, i.e., interstitial fluid (ISF). Specifically, the oxygen permeability of a material commonly employed for peripheral nerve conduit fabrication, collagen, is of the order of 10^–10^ m^2^/s [24]. By comparison, the oxygen permeability of interstitial fluid is reported to be 2.7269 × 10^–9^ m^2^/s [22], approximately an order of magnitude higher than that of collagen. It is noted that the oxygen permeability of CNF is approximately an order of magnitude lower than that of collagen. As such, a range of oxygen permeabilities was selected from 10 times lower (1 order of magnitude ≡ collagen) to 100 times lower (two orders of magnitude ≡ CNF) than ISF. Specifically, the oxygen permeability of the conduit wall was modeled employing values of 10×, 20×, 50×, and 100× less than that of ISF. The resultant oxygen concentration vs. time graphs for each conduit permeability (not shown) were very similar to those presented in Fig. 3; however, it is noted that as the conduit became progressively less permeable, the curves took longer to plateau and plateaued at significantly lower oxygen concentrations. Figure 6 presents the plateau values for oxygen concentration (or time when the concentration went to zero) as a function of nerve to conduit diameter ratio for the 10, 20, 50 and 100 times less than ISF wall permeability values at locations 1, 2 and 3. Figure 6 was generated in a manner comparable to that employed to create Fig. 4.

It is evident from investigation of Fig. 6 that the 10, 20, and 50 times less permeable than ISF wall variants maintained oxygen concentrations greater than zero at all nerve to conduit diameter ratios, at all three locations. Furthermore, it is noted that the oxygen concentrations were consistently lower at all locations at comparable nerve to conduit diameter ratios with progressively decreasing wall permeability. Taken together these two findings suggesting that oxygen diffusion radially across the wall of the conduit does indeed impact concentrations within the conduit and is modulated by the permeability of the wall. The data for the 100 times less than ISF wall permeability variants have oxygen concentrations that trend to 0 mol/m^3^ as the nerve to conduit diameter ratio approaches 1, an observation made at all three locations. As such, for the 100 times less permeable than ISF wall variants, the interior of the conduit is completely hypoxic at all nerve to conduit diameter ratios once the oxygen concentration plateaus, a finding consistent with the CNF modeling results.

It may also be seen from investigation of Fig. 6 that for the 10 times less permeable than ISF wall variants there is little to no effect on the oxygen concentration at a given location as the nerve to conduit diameter ratio increases, suggesting that the wall is so permeable to oxygen that radial diffusion dominates over axial diffusion irrespective of the volume of ISF between the inner wall of the conduit and the nerve stumps. It is noted, however, that the baseline oxygen concentrations at the three locations do follow the expected trend of highest at location 2, where there is no consumption, lowest at location 3, where there is twice the baseline oxygen consumption, and intermediate at location 1. As the wall permeability is decreased to 20 times less permeable than ISF, the oxygen concentration vs. the nerve to conduit diameter curves at locations 1 and 3 (where there is oxygen consumption) trend negatively, indicating that radial diffusion of oxygen across the conduit wall is less dominant, and that axial diffusion in the ISF filled space between the nerve and the inner wall of the conduit becomes progressively more important. Interestingly at location 2 the oxygen concentration remains invariant with nerve to conduit diameter ratio at the 20 times less permeable than ISF wall permeability, an observation potentially attributable to the fact that there is no oxygen consumption at this location making it less susceptible to restricted supply and that radial diffusion remains dominant. A further decrease in wall permeability to 50 times less permeable than ISF results in negative trends in the oxygen concentration vs. nerve to conduit diameter ratio curves at all locations, indicating dominance of axial diffusion over radial diffusion, driven by the restricted oxygen diffusion across the conduit wall. It is noted, however, that radial diffusion of oxygen does occur at this wall permeability value and is critical to maintaining an oxygenated environment within the conduit; a finding exemplified by the hypoxic environment at all locations observed at the 100 time less permeable than ISF wall variants. A summary of the findings derived from Fig. 6 is presented in Table 1. Specifically, the conduit wall permeability and location in the model are paired to provide an overview of the type of diffusion that is dominant.

**Table 1:** Summary of the dominant diffusion regimes

	10×	20×	50×	100×
Location 1	Radial Wall Diffusion	Axial Gap Diffusion	Axial Gap Diffusion	Axial Gap Diffusion
Location 2	Radial Wall Diffusion	Radial Wall Diffusion	Axial Gap Diffusion	Axial Gap Diffusion
Location 3	Radial Wall Diffusion	Axial Gap Diffusion	Axial Gap Diffusion	Axial Gap Diffusion

Regimes observed for paired conduit wall permeabilities (relative to ISF) and locations within the conduit. All combinations apply to conduits of any length in the tested range of 12–16 mm

**Fig. 7:**
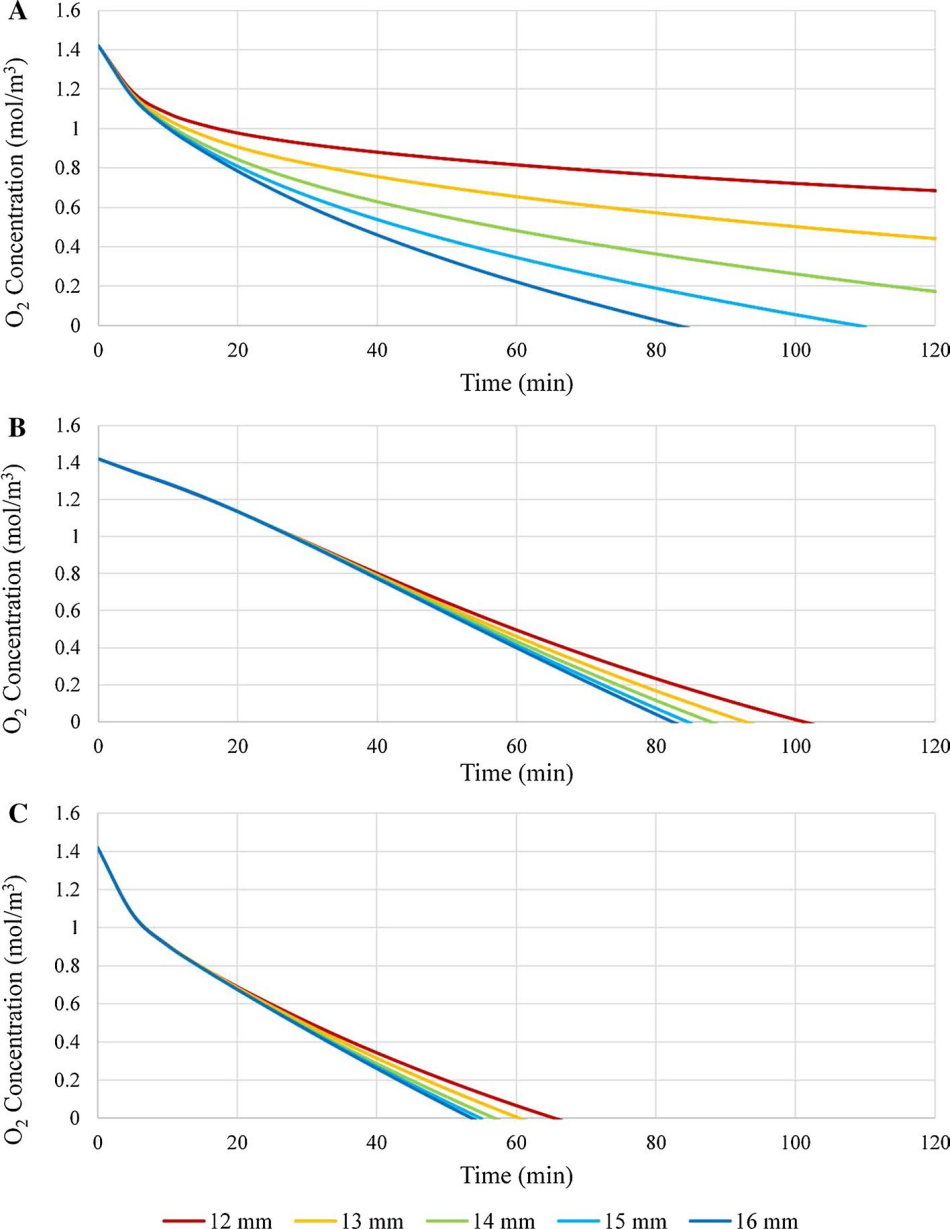
Oxygen concentration vs. time as a function of conduit length (ranging from 12 to 16 mm in 1 mm increments) for a nerve to conduit diameter ratio of 0.7:1 at (**A**) location 1, (**B**) location 2, and (**C**) location 3

Due to animal trial data indicating that for a fixed nerve gap the length of the conduit employed has a significant effect on peripheral nerve regeneration, modeling was performed to explore the effect of conduit length on oxygen concentration and distribution. Five different conduit lengths were tested, ranging from 12 to 16 mm in 1 mm intervals. The base CNF permeability of Table 2 was employed and the model was run until the oxygen concentration reached 0 mol/m^3^. Figure 7 presents the resultant oxygen concentration vs. time data as a function of conduit length for a nominal nerve to conduit diameter ratio of 0.7:1.

**Table 2:** Summary of the parameters used in the COMSOL Multiphysics® model

Parameter	Value
Gap Length	3 mm
Conduit length	15 mm
Nerve diameter	3 mm
Exterior O_2_ concentration	1.4 mol/m^3^
O_2_ diffusion coefficients	
* ISF*	2.7269 × 10^–9^ m^2^/s
* Nerve*	1.7 × 10^–9^ m^2^/s
* Conduit*	1.8 × 10^–13^ m^2^/s
Initial O_2_ concentrations	
* ISF*	1.4 mol/m^3^
* Nerve*	1.4 mol/m^3^
* Conduit*	0 mol/m^3^
O_2_ consumption rate	
* ISF*	0 mol/m^3^-s
* Nerve baseline*	− 0.00092 mol/m^3^-s
* Regenerating*	− 0.00184 mol/m^3^-s
* Conduit*	0 mol/m^3^-s

Investigation of Fig. 7 indicates that as the conduit length increases the time required for the oxygen concentration to reach 0 mol/m^3^ progressively decreases, a trend observed at all three locations and attributable to impaired axial diffusion due to enhanced luminal distances. The data at location 1 show greater dependence of the gradient of the curves at varying conduit lengths relative to those at locations 2 and 3, a fact attributable to the shorter axial diffusion distance for location 1 vs. that at either location 2 or 3 and hence a greater sensitivity of oxygen concentration to conduit length. It is noted that these observations are specific to conditions promoting dominance of axial diffusion over radial diffusion (low nerve to conduit diameter ratio, and low conduit wall permeability). Finally, comparison of the data for the 15 mm conduit (light blue line) of Fig. 7 with the comparable data of Fig. 3 (red line) reveals complete agreement, implying internal consistency of the model.

**Fig. 8:**
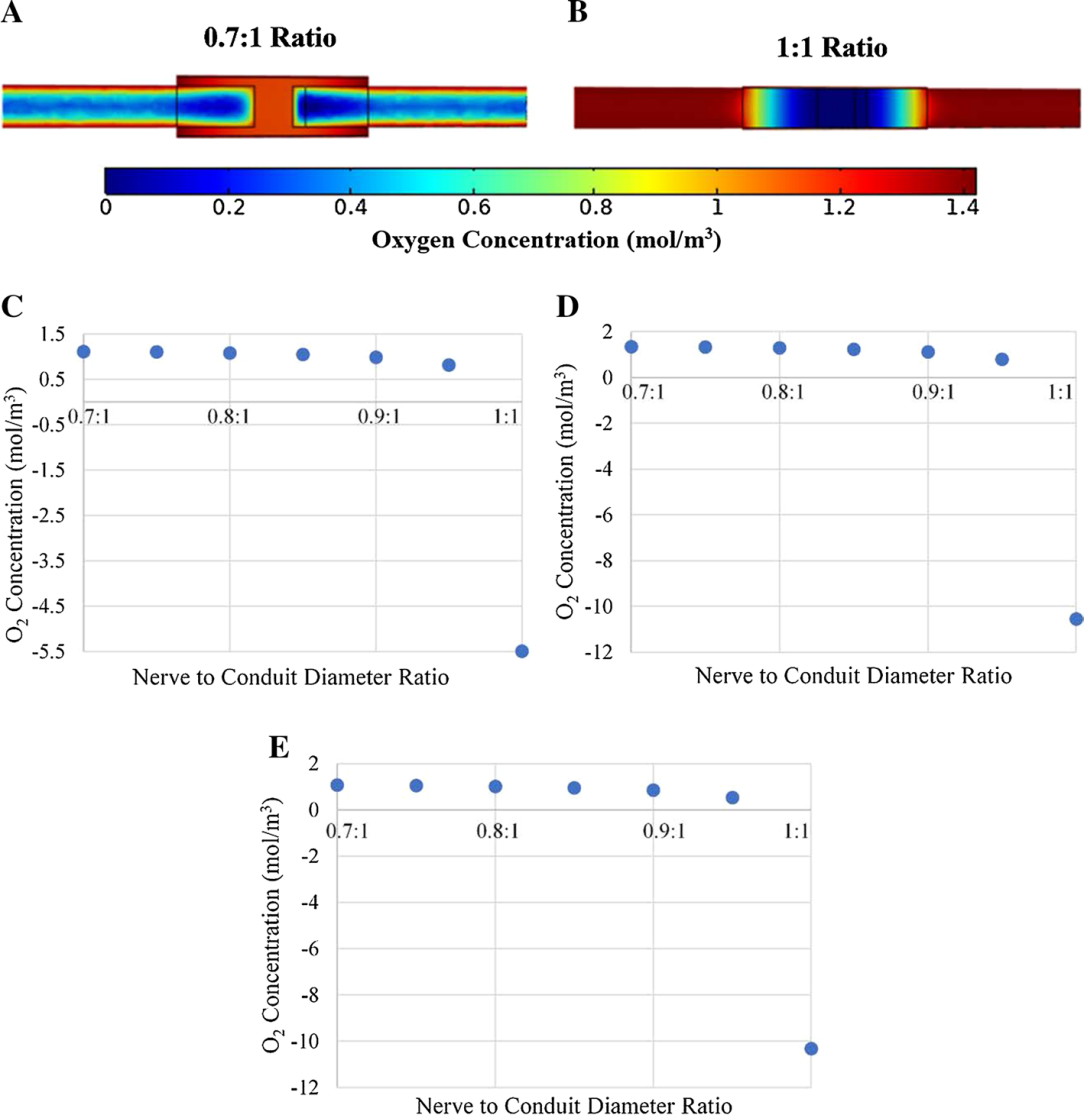
Oxygen concentration plateau profiles for **a** 0.7:1 ratio and **b** 1:1 ratio. Oxygen concentration vs. nerve to conduit diameter ratio for **c** Location 1 **d** Location 2 and **e** Location 3. Note that the conduit wall oxygen permeability was set to zero and the ISF oxygen diffusion coefficient was increased to 100 times its baseline value. Negative oxygen concentration values clearly have no physical meaning but are included to highlight the *r*^2^ dependence of the data

One significant point of interest under conditions such as those above in which axial diffusion is dominant over radial diffusion, is that the trends observed in oxygen concentrations with varying nerve to conduit diameter ratios were typically comparatively linear (or biphasic linear). The observed linearity is somewhat surprising given that the cross section of the model is cylindrical, and as such changes in the radius of the conduit relative to the nerve results in changes in the cross-sectional area of the ISF filled gap by πr^2^. As such one would predict that the relationship between oxygen concentration and nerve to conduit diameter ratios should have an r^2^ dependence in axial diffusion dominant regimes. One potential reason that the observed data did not exhibit a strong *r*^2^ dependence is that the difference between the oxygen diffusion coefficients in ISF and in the nerve itself is very small (see Table 2). To test this hypothesis the conduit wall oxygen permeability was set at zero (to force the system into an axial diffusion dominant regime), and an artificially large difference in oxygen diffusion coefficients for ISF and the nerve were implemented. Specifically, the diffusion coefficient of oxygen through the ISF was increased by a factor of 100, and the nerve diffusivity was maintained at its base value. Setting the parameters as indicated essentially made changes in conduit radii and hence the cross-sectional area of ISF, the sole variable impacting oxygen concentration within the conduit. Figure 8 presents an analysis of oxygen concentration at locations 1, 2, and 3 as a function of nerve to conduit diameter ratio, in both a qualitative and quantitative manner.

Investigation of Fig. 8a indicates that when there is a substantial cross-sectional area of ISF through which oxygen can readily diffuse (0.7:1 nerve to conduit diameter ratio) there is significant oxygen concentration throughout the interior of the conduit. Conversely, when the nerve diameter and the conduit diameter are equal and no cross-sectional area of ISF exists (Fig. 8b), oxygen can only diffuse through the nerve tissue and as a result virtually the entire interior of the conduit is hypoxic with essentially zero oxygen concentration. Figure 8c–e presents the plateau oxygen concentration values as a function of nerve to conduit diameter ratios for locations 1, 2 and 3, respectively. It is evident that at all locations there is only a slight dependence of oxygen concentration on nerve to conduit diameter ratio until a value of approximately 0.9:1, at which point a reverse logarithmic decay trend is observed. It is noted that the final data points of Fig. 8c–e are plotted as negative oxygen concentrations, which hold no physical meaning—however, they are included to illustrate that indeed the model does predict a strong non-linear dependence of oxygen concentration on nerve to conduit diameter ratio under axial diffusion dominant conditions.

## Conclusions

Peripheral nerve injuries are both ubiquitous and debilitating. Fortunately, the human body is capable of regeneration, although for nerve gaps greater than ~ 1 mm surgical intervention is typically required. A common methodology to address peripheral nerve injuries is to suture a conduit over the proximal and distal stumps to facilitate the formation of a microenvironment conducive to regeneration. Despite the prevalence of such surgeries, little is known regarding the microenvironment within the conduit, in particular, the role of oxygen in facilitating peripheral nerve repair is unclear. To provide insight into the concentration and distribution of oxygen with a peripheral nerve conduit, a COMSOL Multiphysics® model of a peripheral nerve injury comprising a 3 mm gap between the proximal and distal stumps was created. The injury site was enclosed within a cellulose nanofiber conduit. The end of the regenerating proximal stump was modeled to consume twice the oxygen per unit time vs. baseline nerve consumption (as per literature). The concentration and distribution of oxygen at three locations within the conduit were analyzed as a function of the nerve to conduit diameter ratio, the permeability of the conduit wall to oxygen, and the length of the conduit.

It was found that the lowest oxygen concentration within the conduit occurred at the end of the proximal stump, where regeneration was occurring. The end of the distal stump had the second lowest oxygen concentration (with baseline oxygen consumption), with the gap in between the stumps having the highest concentration (where no oxygen consumption occurs). Increasing the nerve to conduit diameter ratio (effectively decreasing the ISF filled gap between the nerve and the inner wall of the conduit) led to progressively lower oxygen concentrations for all conduit lengths and locations tested and for conduit wall oxygen permeabilities in accordance with those used in in-vivo studies by the authors and collaborators. The findings were attributed to the dominance of axial diffusion over radial diffusion of oxygen and the decreased ISF filled axial volume as the nerve and conduit diameters approach each other. Increasing the oxygen permeability of the conduit walls to values 10 times less than that of ISF (vs. the baseline case of 100 times less permeable than ISF) resulted in the oxygen concentration at all locations within the conduit being invariant with the nerve to conduit diameter ratio, implying that radial diffusion of oxygen across the conduit wall is the dominant mechanism of oxygen migration for highly permeable conduits. Progressively decreasing the conduit wall permeability led to reversion to the axial diffusion regime at all locations. Increasing the length of the conduit employing the baseline wall permeability demonstrated a consistent decrease in oxygen concentration at all locations within the conduit as the axial distance required for oxygen diffusion increased. The model was shown to be internally consistent, and to follow expected trends with regard to the effect of changes of conduit radii and concomitant change of the ISF filled axial volume on oxygen concentration in an axial-diffusion controlled regime.

## Methods

**Fig. 9:**
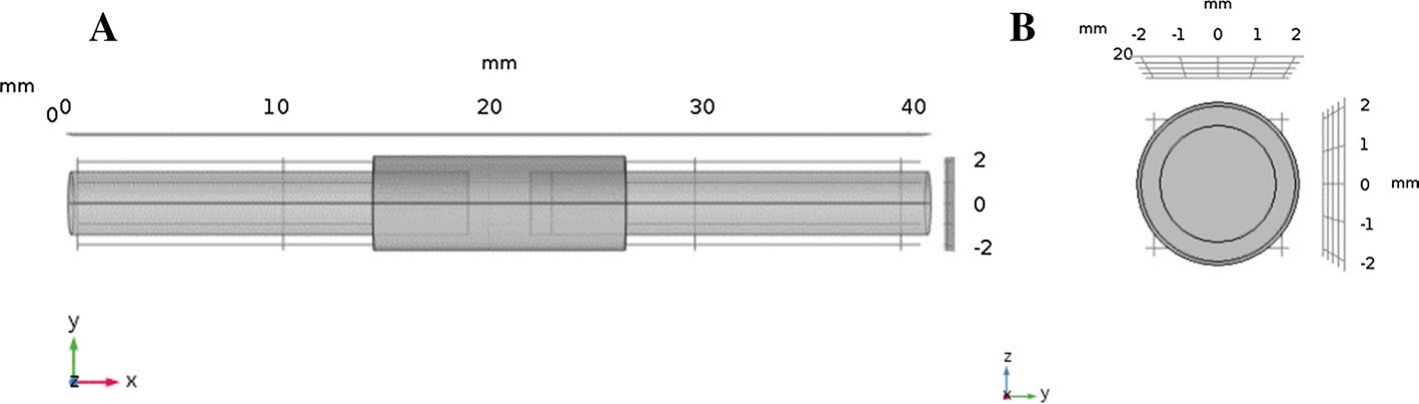
**a** x–y view of the conduit bridging the nerve gap. **b** y–z view of the model looking down the axis of the nerve, the inner circle represents the cross section of the nerve, the middle layer corresponds to ISF, and the thin outer layer represents the conduit

A model of the severed peripheral nerve/conduit system was created in the software package COMSOL Multiphysics®. The model integrates material properties of the conduit with in-vivo conditions internal and external to the conduit to emulate the post-surgical environment. The model, presented in Fig. 9, includes an outer hollow cylinder which represents the conduit (variable length, diameter, and permeability of the wall to oxygen), and two inner solid cylinders which represent the proximal (right side of Fig. 9) and distal (left side of Fig. 9) nerve stumps (3 mm diameter). The boundaries of the model, that is the end of the nerves external to the conduit, were set to be impermeable to the inflow of oxygen to mimic the nerve extending for considerable distances within the body. The volume in between the nerve segments (3 mm in length), and the volume between the nerve stumps and the inner conduit wall (variable), were modeled to comprise interstitial fluid (ISF).

As may be seen in Fig. 9a, a 1 mm section at the end of the proximal stump is delineated, and in accordance with the findings of Lim et al., is modeled to be regenerating with a resultant oxygen consumption twice that of the remainder of the nerve.

The package “Transport of Diluted Species” was implemented within COMSOL Multiphysics® to appropriately represent oxygen diffusion coefficients for the various media of the model, and to generate concentration profiles even at comparatively low numerical values. Since most of the quantitative research regarding oxygen concentration during nerve regeneration has been performed employing murine models, the model was built using data reported for mice. The base parameters used for modelling are summarized in Table 2 and are discussed below.

Values for the nerve diameter, nerve gap length, conduit length and conduit diameter were chosen based on the experimental murine studies performed by the authors and their collaborators. The remaining parameters were determined from literature. Specifically, the base oxygen concentration in the ISF was taken to be that of the average partial pressure of oxygen in arterioles (P_O2_ = 26.4 mmHg) [21] and was assumed to be constant, since the vasculature continuously replenishes it. The oxygen diffusion coefficient for ISF was estimated to be equivalent to that for water and was obtained by extrapolating data of oxygen diffusion coefficient vs. temperature reported by Han et al. (1996) to physiologic temperature. The oxygen diffusion coefficient through mouse nerve tissue was assumed to be equivalent to that through rat peripheral nerve tissue as reported by Lagerlund et al. [23]. Oxygen consumption rates were based on the premise that the only entities consuming oxygen in the system were the nerve stumps, and that consumption was through both normal metabolic processes [22], and through active repair mechanisms [20]. The two studies [20, 22] provided values for baseline metabolic consumption that were almost identical (with a few percent), the higher value was adopted for use in the present work.

A critical parameter of the model is the oxygen permeability of the CNF conduit, a value not reported in the literature under relevant humidity conditions (100%). As such, to determine the oxygen permeability of the CNF conduit, three CNF films were prepared using a previously employed thin film casting method and measured for oxygen permeation employing a Mocon OX-TRAN Model 22/2 (L). A measurement at 100% relative humidity (RH) was not within the capabilities of the instrument, as such permeation measurements were made at a range of RH values (50, 80 and 90%) and the data extrapolated to 100% RH. Extrapolation was performed by plotting RH on the *x*-axis and inverse permeation on the *y*-axis, a linear trend line was subsequently fit with an R^2^ value of 0.969 and an extrapolated permeation rate of 1.41E-13 m^2^/s. When replotted with the calculated value, the graphical trend of permeation rate and its dependence on RH revealed that the calculated value was likely a low estimate. Secondary extrapolation of the data was, therefore, performed using the data points from the 80 and 90% RH measurements to calculate a permeation rate of 2.22E-13 m^2^/s with a R^2^ value of 1.00, creating a high-end estimate of the permeation rate. Having established a high and a low estimate of the value of the permeation rate, a value in the middle of the range was adopted for the purpose of the model.

**Fig. 10:**
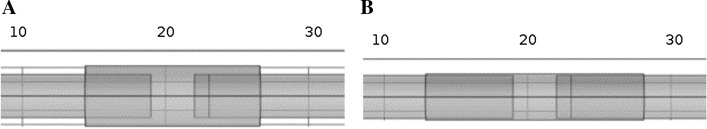
Two variations of the CNF conduit model. **a** 12 mm long conduit with a 0.7:1 nerve to conduit diameter ratio and **b** 15 mm long conduit with a 1:1 nerve to conduit diameter ratio (no radial gap). Note that the diameter, length and relative placement of the proximal and distal nerve stumps have remained constant; however, the ends of the nerves are not shown to show the conduit section more clearly

The dimensions of the model, specifically the ratio of nerve to conduit diameter and the conduit length, were adjusted sequentially to analyze their effect on oxygen diffusion. The ratio of nerve to conduit diameter was varied between the following values: 1:1, 0.95:1, 0.9:1, 0.85:1, 0.8:1, 0.75:1, and 0.7:1. To create the different ratios of nerve to conduit diameter, the nerve diameter was kept constant (at 3 mm), while the conduit diameter was varied. Equation 1 presents a proportion that was employed to calculate conduit diameter X for a given nerve to conduit diameter ratio R. In addition, conduit length was varied between 12 and 16 mm, in 1 mm increments. Variation of the length of the conduit was accomplished by centering the conduit over the middle of the gap between the proximal and distal nerve stumps. Figure 10 presents, in schematic form, the difference between a 0.7:1 and a 1:1 nerve to conduit diameter ratio, in addition to the difference between a 12 mm and a 15 mm long conduit.

1 $$\frac{R}{1} = \frac{{3~\;{\text{mm}}}}{{X~\;{\text{mm}}}}$$

Equation 1. Proportion relating the ratio (*R*) of the nerve (3 mm) to conduit diameter (*X*). Nerve diameter is constant, ratio is controlled, and conduit diameter is solved for.

**Fig. 11:**
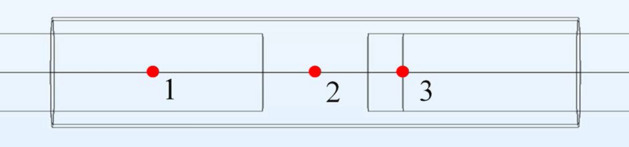
Three locations where oxygen concentration vs. time data were monitored. Note the ends of the nerves are not shown in this figure

The COMSOL Multiphysics® model generated oxygen concentration profiles that could be analyzed both spatially and temporally and in a quantitative and qualitative manner. The quantitative data generated was in the form of oxygen concentration vs. time at given spatial positions, while the qualitative assessment provided spatial mapping of oxygen concentration employing a colorimetric scale. To collect representative data three positions within the conduit were selected; at the center of the distal stump (position 1), the center of the gap between the nerve stumps (position 2), and at the center of the proximal stump at the intersection of the regenerating and baseline sections (position 3), see Fig. 11.

Location 1 was chosen as a representation of a region with normal, baseline oxygen consumption by a nerve. Location 2 is in the center of the nerve gap, and in the center of the conduit. In location 2 there is no direct oxygen consumption by the nerves (due to their absence); however, oxygen could potentially be depleted from this region due to consumption by the two neighboring nerve stumps. Location 3 was selected, because initial testing indicated that the interface between the regenerating nerve and the baseline proximal stump consistently had the lowest oxygen concentration in the system. Analysis of the oxygen concentration at each location as a function of time provided insight into how oxygen diffused into the luminal space, and how variation of parameters altered concentration profiles.

## Data Availability

Available from the corresponding author upon request.

## References

[1] Gaudet AD, Popovich PG, Ramer MS (2011). Wallerian degeneration: Gaining perspective on inflammatory events after peripheral nerve injury. J Neuroinflamm.

[2] Christie KJ, Zochodne D (2013). Peripheral axon regrowth: New molecular approaches. Neuroscience.

[3] Ichihara S, Inada Y, Nakamura T (2008). Artificial nerve tubes and their application for repair of peripheral nerve injury: an update of current concepts. Injury.

[4] Muheremu A, Ao Q (2015). Past, present, and future of nerve conduits in the treatment of peripheral nerve injury. Biomed Res Int.

[5] Barton MJ, Morley JW, Stoodley MA, Lauto A, Mahns DA (2014). Nerve repair: toward a sutureless approach. Neurosurg Rev.

[6] Kehoe S, Zhang XF, Boyd D (2012). FDA approved guidance conduits and wraps for peripheral nerve injury: a review of materials and efficacy. Injury.

[7] Mobini S, Spearman BS, Lacko CS, Schmidt CE (2017). Recent advances in strategies for peripheral nerve tissue engineering. Curr Opin Biomed Eng.

[8] Evans PJ, Midha R, Mackinnon SE (1994). The peripheral nerve allograft: a comprehensive review of regeneration and neuroimmunology. Prog Neurobiol.

[9] Braga Silva J, Marchese GM, Cauduro CG, Debiasi M (2017). Nerve conduits for treating peripheral nerve injuries: a systematic literature review. Hand Surg Rehabil.

[10] Rinker B, Vyas KS (2014). Clinical applications of autografts, conduits, and allografts in repair of nerve defects in the hand: Current guidelines. Clin Plast Surg..

[11] Kokai LE, Lin YC, Oyster NM, Marra KG (2009). Diffusion of soluble factors through degradable polymer nerve guides: controlling manufacturing parameters. Acta Biomater.

[12] Shim S, Ming GL (2010). Roles of channels and receptors in the growth cone during PNS axonal regeneration. Exp Neurol.

[13] Aydin A, Özden BÇ, Karamürsel S, Solakoǧlu S, Aktaş Ş, Erer M (2004). Effect of hyperbaric oxygen therapy on nerve regeneration in early diabetes. Microsurgery.

[14] Oroglu B, Turker T, Aktas S, Olgac VAM (2011). Effect of hyperbaric oxygen therapy on tense repair of the peripheral nerves. Undersea Hyperb Med.

[15] Cho Y, Shin JE, Ewan EE, Oh YM, Pita-Thomas W, Cavalli V (2015). Activating injury-responsive genes with hypoxia enhances axon regeneration through neuronal HIF-1α. Neuron.

[16] Yao C, Shi X, Zhang Z, Zhou S, Qian T, Wang Y (2016). Hypoxia-induced upregulation of miR-132 promotes Schwann cell migration after sciatic nerve injury by targeting PRKAG3. Mol Neurobiol.

[17] Haapaniemi T, Nylander G, Kanje M, Dahlin L (1998). Hyperbaric oxygen treatment enhances regeneration of the rat sciatic nerve. Exp Neurol.

[18] Shams Z, Khalatbary AR, Ahmadvand H, Zare Z, Kian K (2017). Neuroprotective effects of hyperbaric oxygen (HBO) therapy on neuronal death induced by sciatic nerve transection in rat. BMC Neurol..

[19] Sanchez EC (2007). Hyperbaric oxygenation in peripheral nerve repair and regeneration. Neurol Res.

[20] Lim TKY, Shi XQ, Johnson JM, Rone MB, Antel JP, David S (2015). Peripheral nerve injury induces persistent vascular dysfunction and endoneurial hypoxia, contributing to the genesis of neuropathic pain. J Neurosci.

[21] Torres Filho IP, Leunig M, Yuan F, Intaglietta M, Jain RK (1994). Noninvasive measurement of microvascular and interstitial oxygen profiles in a human tumor in SCID mice. Proc Natl Acad Sci USA.

[22] Han P, Bartels DM (1996). Temperature dependence of oxygen diffusion in H_2_O and D_2_O. J Phys Chem.

[23] Lagerlund TD, Low PA (1987). A mathematical simulation of oxygen delivery in rat peripheral nerve. Microvasc Res.

[24] Cheema U, Rong Z, Kirresh O, Macrobert AJ, Vadgama P, Brown RA (2012). Oxygen diffusion through collagen scaffolds at defined densities: Implications for cell survival in tissue models. J Tissue Eng Regen Med.

